# Medicine in spine exercise (MiSpEx) for nonspecific low back pain patients: study protocol for a multicentre, single-blind randomized controlled trial

**DOI:** 10.1186/s13063-016-1645-1

**Published:** 2016-10-20

**Authors:** Daniel Niederer, Lutz Vogt, Pia-Maria Wippert, Anne-Katrin Puschmann, Ann-Christin Pfeifer, Marcus Schiltenwolf, Winfried Banzer, Frank Mayer

**Affiliations:** 1 Department of Sports Medicine, Goethe University Frankfurt, Ginnheimer Landstraße 39, 40487 Frankfurt am Main, Germany; 2 University Outpatient Clinic, Centre of Sports Medicine, University of Potsdam, Potsdam, Germany; 3 Department of Orthopaedics and Trauma Surgery, Heidelberg University Hospital, Heidelberg, Germany; 4 Department of Sociology of Physical Activity and Health, Cluster of Excellence in Cognitive Sciences, University of Potsdam, Potsdam, Germany

**Keywords:** Sensorimotor training, Motor control, Low back pain, Exercise, Functional capacity, Individualized intervention

## Abstract

**Background:**

Arising from the relevance of sensorimotor training in the therapy of nonspecific low back pain patients and from the value of individualized therapy, the present trial aims to test the feasibility and efficacy of individualized sensorimotor training interventions in patients suffering from nonspecific low back pain.

**Methods and study design:**

A multicentre, single-blind two-armed randomized controlled trial to evaluate the effects of a 12-week (3 weeks supervised centre-based and 9 weeks home-based) individualized sensorimotor exercise program is performed. The control group stays inactive during this period. Outcomes are pain, and pain-associated function as well as motor function in adults with nonspecific low back pain. Each participant is scheduled to five measurement dates: baseline (M1), following centre-based training (M2), following home-based training (M3) and at two follow-up time points 6 months (M4) and 12 months (M5) after M1. All investigations and the assessment of the primary and secondary outcomes are performed in a standardized order: questionnaires – clinical examination – biomechanics (motor function). Subsequent statistical procedures are executed after the examination of underlying assumptions for parametric or rather non-parametric testing.

**Discussion:**

The results and practical relevance of the study will be of clinical and practical relevance not only for researchers and policy makers but also for the general population suffering from nonspecific low back pain.

**Trial registration:**

Identification number DRKS00010129. German Clinical Trial registered on 3 March 2016.

**Electronic supplementary material:**

The online version of this article (doi:10.1186/s13063-016-1645-1) contains supplementary material, which is available to authorized users.

## Background

Nonspecific low back pain is the most common kind of musculoskeletal pain with a lifetime prevalence of up to 90 % [1, 2] and of particular relevance in industrialized countries. Point prevalence varies between 30 and 50 %, depending on age and sex [1]. When focusing on competitive elite athletes only, actual research findings estimate the lifetime prevalence at 60 % and the point prevalence at 18 % [3, 4]. Just as in the general public, prevalences vary in different subsamples of athletes, depending on the specific sport, age or sex [5, 6].

Neuromuscular and/or structural deficits [7, 8], mostly accompanied by psychological or social factors (‘yellow flags’) [9], are known risk factors for both the onset and chronification of nonspecific low back pain. With valuable impact on these factors and the back pain itself, target-oriented movement training is of increasing relevance in (secondary) prevention and therapy [10, 11]. Evidence-based therapeutic approaches have changed and are no longer solely passive-reactive but multidisciplinary-active with a major physical activity part. However, the evidence for the effectiveness of physical activity in the therapy of nonspecific low back pain is mixed. A recent meta-analysis indicates that physical activity has positive effects in the therapy of nonspecific low back pain [10]. In addition, the authors show that, using pooled subgroup effects, coordinative and stabilizing training also has an effect by addressing sensory deficiencies. The latest Cochrane review, however, only provides low to moderate quality evidence that isolated motor control exercises are able to improve pain in the short, intermediate or long term [11]. The authors conclude that a final proof for the efficacy of motor control exercise on relevant functional factors like kinematics, strength, postural control and pain is still missing.

A further potential explanation for the lack of consistent evidence for the beneficial impact of physical activity on nonspecific back pain may be that the impact of physical activity on pain is moderated by contextual and individual psychological and social factors (so-called yellow flags [12]). A better understanding of the role of these moderating factors is crucial to evaluate the role of physical activity for the treatment of chronic back pain and eventually enable the personalization of such physical activity interventions. Individualizing the sensorimotor therapy with respect to individual skill level, demands, preferences, and potential moderating factors such as pain, physical activity, pain experience, stress, life context and the medical care context may lead to greater therapy success [12]. Likewise, determining the optimal dose for treating patients as individuals is still a matter of debate [13, 14].

Against the background of the need for further evidence for the efficacy or non-efficacy of motor control exercise targeting neuromuscular factors like kinematics, strength, postural control and pain [11], the purpose of this study is to test (1) the feasibility and (2) the efficacy of individualized motor control interventions in nonspecific low back pain patients. We hypothesize that individualized motor control exercises lead to an improvement in motor function, assessed as maximal back extension torque, in low back pain patients and in comparison to a control group. Secondary research questions are: (1) does individualized motor control exercises lead to an improvement in motor function, pain and/or pain-related variables in low back pain patients and compared with a control group at short and/or long term and (2, exploratory) to what extent do moderating factors play a role in non-responder detection dose-response considerations and therapy individualization?

## Methods/Design

### Study design and flow

The study consists of a multicentre (Appendix A), single-blind two-armed randomized controlled trial to evaluate the effects of a 12-week individualized sensorimotor exercise program on pain, pain-related outcomes and function in nonspecific low back pain adults compared with a waiting control group. The study has been approved by the independent Ethics Committee of the University of Potsdam (committee’s reference number 3/2016; amendment for number 47/2013). Date of preliminary approval was the 25 January 2016; date of the final approval was the 9 May 2016. The study was planned and is performed in agreement with the Declaration of Helsinki (Version Fortaleza 2013). Trial registration number is DRKS00010129 (DRKS, German Clinical Trials Register, drks.de; 3 March 2016). All adverse events will be reported.

Primary outcome is trunk extension strength [Nm]. The assessment and analyses of all records are performed by blinded evaluators. Five assessment visits (M1-M5) will take place over a period of 12 months.

### Participants

#### Sample size determination

A pilot feasibility study was conducted (unpublished material). The therein found outcome with the largest effect size was selected as the primary outcome for the present study. Thus, primary outcome is maximal back extension torque. Sample size calculation was based on the effect size of d = 0.24 (based on the changes in primary outcome from baseline to post-intervention (12 weeks)). For the main study, effect size was conservatively completed to d = 0.2.

Participants are allocated to intervention or control group at the ratio of 2:1. Normal distribution and variance equality are assumed. Following a determined α-error probability of 2.5 % and a β-error probability of 0.1, a minimal sample size of 1186 patients are consequently to be included into analysis (*n* = 791 in the intervention and *n* = 395 in the control group). As a dropout rate of 30 % is assumed, a total of *N* = 1542 participants are included in the study. Although motor control and stabilizing exercise are likely to show equal effects for the general population and for top athletes [15], medical access and the potential to implement medical exercise regimes into everyday life differ between these populations (e.g. training schedule, competitions). According to study aim 1, and beside the studies’ main target group, we make additional effort to also recruit a sample of athletes to gain further (beyond the main analyses) insight and test the feasibility in this much less investigated population. For dichotomization purpose, only top competitive athletes (A to D pool, participating in top level international, federal or state competitions) are further treated as athletes.

#### Recruitment, screening and informed consent

Representative volunteers are recruited at clinical low back pain consultation hours, through flyers, local newspapers and bulletins, and personal recruitment. Athletes are further recruited by personal approach in one of the participating study sites, mostly at the mandatory annual sports medical examination. Only general information is provided during recruitment. Interested persons are scheduled for a visit in one of the six study sites and then screened for eligibility. Males and females aged 18–65 years with chronic low back pain are considered eligible if they fulfil the following inclusion criteria: at least one episode (≥ 4 days) of nonspecific back pain in the last 12 months, ability to understand the extent and meaning of the study and to answer a questionnaire without help.

Exclusion criteria consist of acute infection, pregnancy, inability to execute a one-legged stance, diseases with contraindication for exercise (including spine complaints based on space-occupying, inflammatory, traumatic or systemic processes [12]) and change or progression in the severity, localization or type of back pain during the last 7 days. Following application of inclusion and exclusion criteria, patients are approved by the physician in charge. Each participant signs informed consent prior to study enrolment.

#### Randomization procedure and blinding

After inclusion and completing the first visit, volunteers are block-randomised (n_block_ = 18; 2:1 basis) into either the intervention or control group by the study coordinator. Randomization order follows the study inclusion order. Each study site receives its own randomization lists once a week from the primary study centre; the randomization sequence is generated using a computer-based algorithm (www.randomization.com). All assessors and study personnel other than the study coordinator (and the therapist) are blinded. Participants are told not to communicate their group allocation to other participants or study staff.

### Experimental procedure

**Fig. 1:**
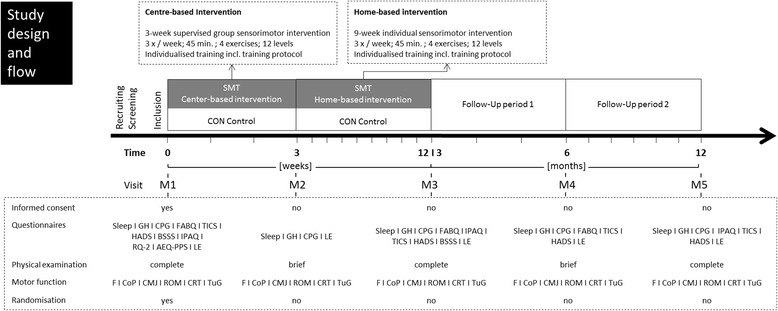
Study time bar and flow charts. All participants are scheduled to five visits with standardized assessment procedures. Subjects are 2:1 randomized into sensorimotor training (SMT) and control (CON) group for centre-based (3 weeks) and home-based phase (9 weeks). *M1-M5* visit measurement number 1 to number 5. Questionnaires: *CPG* Chronic Pain Grade questionnaire, *FABQ-D* Fear-Avoidance Beliefs Questionnaire, *TICS* Trier Inventory for Chronic Stress, *HADS-D* Hospital Anxiety and Depression Scale, *BSSS* Berlin Social Support Scales, *IPAQ* International Physical Activity Questionnaire, *RQ-2* the Relationships Questionnaire 2, *AEQ-PPS* Avoidance Endurance Questionnaire, *SES* socioeconomic status, sleep, *GH* general health, *LE* life events, motor function: *F* force/strength/torque, *CoP* centre of pressure; static balance, *CMJ* counter movement, *ROM* range of motion/kinematic analyses, *CRT* Chair Rise Test, *TuG* Timed Up and Go Test

In this multicentre intervention trial, a 12-week sensorimotor intervention is performed (3 weeks supervised centre-based followed by 9 weeks home-based) after the randomization (intervention group only). The control group does not receive any (additional) therapeutic or placebo treatment during this period. Each participant is scheduled for five measurement visits (M1-M5) prior to (M1) and following centre-based (M2), following home-based training (M3) and at two follow-up points in time (M4: 6 months after M1 and M5: 12 months after M1). All investigations and thus the assessment of the primary and secondary outcomes are performed in a standardised order: questionnaires – clinical examination – biomechanics. Whilst all biomechanical assessments (including the primary outcome) are assessed at each visit, the clinical examination and questionnaires are assessed in a sequenced order. The experimental procedure is shown in detail in Fig. 1 and Additional file 1. Although the measurement periodization is defined, an individual tolerance time of 7 days (M2, M3), 14 days (M4) and 30 days (M5) is set.

#### Questionnaires – assessment of back pain and moderating factors

At each measurement day (M1-M5) a psychometric assessment is performed to detect the moderating factors (‘yellow flags’) for the chronification of back pain. The test battery will be rotated (Fig. 1) and is partially based on subscales in order to reduce the burden for participants. Pain is assessed by the Chronic Pain Grade questionnaire (CPG) [16], which allows a differentiation in the subscales pain intensity (PI: 0 = “no pain” to 100 = “the worst pain imaginable”), and disability (DS: 0 = “no disability” to 100 = “I was incapable of doing anything”) in the past 3 months. Furthermore, participants can get classified into one of the four hierarchical pain and disability grades ranging from low pain/disability (grade I) to high pain/disability scores (grade IV) [17].

The five moderating factors were assessed using the following psychometric instruments: (1) physical activity: International Physical Activity Questionnaire (IPAQ) [18] and type of physical activity. (2) Pain experience: pain-related cognitions by the Fear-Avoidance Beliefs questionnaire (FABQ-D, [19]), avoidance‐endurance‐behaviour by the Avoidance-Endurance Questionnaire (AEQ; [20] and anxiety and depression by the Hospital Anxiety and Depression Score (HADS-D; [21]) ). (3) Stress: chronic stress by the Trier Inventory of Chronic Stress (TICS; [22]) and critical life events in the last 3 months. (4) Personal life context: social support by the Berlin Social Support Scales (BSSS; [23], individual attachment style by the Relationship Questionnaire 2 (RQ‐2; [24, 25]) as well as the socioeconomic status (CASMIN; [26] and lifestyle (alcohol, smoking, general health and sleeping status). (5) Medical care context: health services options (see also DSF, [27]) .

### Physical examination

The medical/physical examination has two aims: first, secondary outcome variables are assessed; second, contraindications for the following motor function assessment are screened.

At M1, M3 and M5, the complete medical assessment, according to the expert consensus standards of the German Association for Sports Medicine and Prevention [28] are performed by a trained sports medicine physician. This examination consists of (1) a complete medical anamnesis, (2) a training anamnesis, and (3) a general-internal and an orthopaedic examination. At M2 and M3, only a brief anamnesis to assess potential clinically relevant changes is conducted. In case of potential relevant changes, a complete medical assessment is performed at M2 and M3 as well.

### Motor function

Following the physical examination and in case of no contraindications, functional outcomes are assessed in a standardized order at each visit. Following consensus discussions and in order to prevent muscle fatigue prior to torque assessment, the order is as follows: (1) posturography, (2) jumping assessment, (3) kinematics, (4) clinical testing, (5) strength. If a functional assessment is done for both the left and right side, side order will be randomized. Randomization sequence is generated using a computer-based algorithm (www.randomization.com). For standardizing purpose (setting and device familiarization) and to avoid bias, one demonstration (by the investigator) and one test run are completed before each new biomechanical outcome assessment start. A numerical rating scale (NRS, 0–10 points Likert scale) is used to assess actual pain prior to and following the motor function assessment.

#### Posturography

Postural control in single and bipedal stance is assessed using balance boards (Wii Balance Board, Nintendo, Kyoto, Japan; modified by CSMi Computer Sports Medicine Inc., Stoughton, MA, USA). Postural sway is estimated by the trace length [mm] of the excursion of the centre of pressure (COP). For the measurement, participants were asked to maintain in upright stance as still as possible for 30 seconds without wearing shoes, with their hands on their hips and their eyes open, standing on two balance boards. Afterwards, the single leg stands (left and right) followed in the randomized order (each 30 s, eyes open, one single balance board) with the contralateral knee bend (90°).

In comparison to a laboratory-grade force platform and with an intraclass correlation (ICC) of 0.81 (single limb stance, 95 % CI 0.39–0.92) and 0.77 (double limb stance, 95 % CI 0.69–0.95), respectively, the Wii balance board shows a sufficient validity [29]. Accordingly and with an ICC of 0.89 (95 % CI: 0.76–0.95) for single limb balance assessments and an ICC of 0.86 (95 % CI: 0.54–0.9) for bipedal stance, a high reproducibility was shown [29].

#### Jumping assessment

Jumping ability is afterwards determined using the same balance boards. Countermovement jumps (CMJ) and combined CMJs followed by reactive jumps are executed. Whilst each participant performs double leg jumps, single leg jumps are, for security purpose, performed by athletes only.

After warm-up (30 s tapping on a stepper with a 14–21 cm high) and familiarization, participants perform two bipedal CMJs, followed by two combined bipedal CMJs/reactive jump. For the reactive jumps, all participants are told to immediately add another jump after landing from the CMJ. All athletes afterwards jump twice with the left and twice with the right leg (randomised order), each time starting with a CMJ followed by a CMJ with a reactive jump. Main outcomes will be jumping height in cm for the CMJ and contact time [ms] for the reactive jumps.

The balance board is able to precisely (*r* = 0.99 in comparison to gold standard laboratory-grade force platform) measure vertical ground reaction force and thus jumping performance [30]. Data from both the posturography and the jumping tests are stored for the later analysis using the HUMAC Balance program (CSMi Computer Sports Medicine Inc., Stoughton, MA, USA).

#### Kinematics

A non-invasive external three-dimensional wireless movement analysis system (IMU sensors, Ergotest Innovations, Porsgrunn, Norway) with a 200 Hz sample rate is used to collect kinematic data. Sufficient test–retest reliability of this measurement system for lumbar spine kinematics (*r* ^2^ > 0.97; RMSE = 4.1°; *p* < 0.001) has recently been demonstrated [31]. Six sensors are attached, two on the lower back (each 5 cm left/right from L3), two on the pelvis (left and right iliac spines), and two on the thigh, each 10 cm above the medial tibia plateau. Participants perform over the maximal range of motion in a self-determined and comfortable velocity with eyes open. After familiarization trial and device zeroing, all subjects perform two times maximal spine flexion-extension movements, followed by maximal lateral flexion movements (two times), followed by two maximal rotation movements (side order randomised). Maximal ranges of motion (ROMs) [°] will be selected for further analysis.

Data were stored on a data processor for the following offline analysis. Maximal ROM was computed from raw data using MuscleLab Professional (Ergotest Innovations, Porsgrunn, Norway).

#### Clinical testing

The Timed Up and Go test (TuG) for mobility testing and the Chair Rise Test (CRT) for lower extremity strength/force are performed during clinical function testing [32]. For the TuG, subjects will sit on a chair with arm support, stand up, walk straight 3 m to a mark on the floor, turn around, walk back to the chair and sit down again. Participants then (CRT) are set on a chair (46 cm sitting height, arms crossed in front of the breast) and are asked to stand-to-sit five times as fast as possible without losing control.

Time [s] is the outcome further analysed for CRT and TuG; common stopwatches are used to assess it. Both tests are performed twice; the best result will be selected for further analysis.

#### Torque/strength

Maximum trunk extension strength assessment with isokinetic or, depending on the participating centres’ isokinetic device availability, isometric maximum trunk extension torque testing [Nm] is considered to be the primary outcome. To obtain maximal torque (isokinetic, *n* = 4 study sites) or maximum isometric voluntary force (isometric, maximal isometric voluntary force (MIVF), *n* = 2 study sites) of the trunk extensors (primary outcome) and flexors, validated devices from various but nevertheless renowned producers are used.

After a specific warm-up with 30 isokinetic repetitions at 60°/s (isometric: two submaximal practice trials), five (three) tests at 60°/s (3 s) were performed, separated by 2-min rest intervals. Ranges of motions (isokinetic) are device-specific set to 40–45° in flexion and 10–15° in extension. Subjects were verbally encouraged in a standardized way to elicit maximal effort. Collected data were immediately analysed and the highest torque (force) was considered to be representative for further statistical analysis.

### Intervention

#### Therapy flow and phases

All interventions are guided (centre-based) or instructed (home-based) by trained and experienced medical training or sports therapists. Following the randomization at the end of M1, intervention group participants are scheduled to a 3-week centre-based guided intervention, followed by a 9-week home-based individual training. In each phase and in accordance with the guidelines [33], intervention participants are told to train three times a week with a mean duration of 30 minutes. The program consists of four different sensorimotor exercises. For individualization purposes, all exercises comprise 12 different levels and offer the possibility to add self-initiated additional motor tasks like ball tapping (single-handed, on the floor or against the wall and/or additional weight). Two of the exercises are dedicated to directly train the core stabilizing and/or core surrounding muscles whilst the two other exercises are considered to impact indirectly on the upper and/or lower extremities. The exercises are commonly described as: (1) quadrupedal/all-fours stability; (2) deadlift/rowing; (3) double leg–single leg heel-pad stance; (4) side planks. Exercises will be performed with three series of ten repetitions each. In between series, a 2-minute break (self-controlled) is held. Details on the intervention and belonging levels are provided in Table 1.

**Table 1:** Interventional exercises details. For each exercise, level (1–12), surface (stable/instable) and description are provided

Exercise 1: quadrupedal/all-fours stability		Exercise 2: deadlift/rowing		Exercise 3: double leg – single leg heel-pad stance		Exercise 4: side planks	
Stable ground	Instable ground	Stable ground	Instable ground	Stable ground	Instable ground	Stable ground	Instable ground
1. Hand and knee stance: Bending, stretching a leg 2. Hand and knee stance diagonal arm and leg: from body centre upwards (horizontal) 4. Hand and feet stance: bending, stretching a leg	3. Hand and knee-stance diagonal arm and leg: from body centre upwards (horizontal) 5. Hand and feet stance: bending, stretching a leg 6. Hand and feet-stance: diagonal arm and leg: from body centre upwards (horizontal) 7. Hand and feet-stance: release arm, trunk rotation 8. Planks: leg horizontal 9. Planks: diagonal leave arm and leg 10. Planks: leave arm, rotate trunk 11. Planks: leave arm and diagonal leg, rotate trunk 12. Press-up: leave arm	1. Rowing plus additional weight 3. Rowing in ball stance plus additional weight 5. One handed rowing plus additional weight 6. One handed rowing plus additional weight In ball stance 9. One handed rowing plus additional weight in single leg stance 10. One handed rowing plus additional weight In single leg ball stance	2. Rowing plus additional weight 4. Rowing in ball stance plus additional weight 7. One handed rowing plus additional weight 8. One handed rowing plus additional weight In ball stance 11. One handed rowing plus additional weight In single leg stance 12. One handed rowing plus additional weight In single leg ball stance	1. Bipedal: heel-pad-stance 3. Unipedal stance plus hip abduction 4. Unipedal stance plus hip abduction and leg extension 6. Unipedal ball stance plus hip abduction and leg extension 10. Unipedal squat 11. Unipedal squat plus additional weight	2. Bipedal: heel-pad-stance 5. Unipedal stance plus hip abduction and leg extension 7. Unipedal ball stance plus hip abduction and leg extension 8. Squat in ball stance 9. Squat in ball stance and hip bending 12. Squat in ball stance with additional weight	1. Knee on ground; hip released from ground 2. Knee on ground; hip stable 3. Knee on ground; hip up/down 5. Legs stretched, hip fixed upwards 8. Legs stretched, release leg from ground 10. Legs stretched, release leg and diagonal arm from ground: horizontal-contact 12. Legs stretched, hip upwards, release leg and diagonal arm from ground: horizontal-contact	4. Knee on ground; hip up/down 6. Legs stretched, hip fixed upwards 7. Legs stretched, hip up/down 9. Legs stretched, release leg from ground 11. Legs stretched, release leg and diagonal arm from ground: horizontal-contact

#### Individualization

For individualization purposes, the level (including the number and type of self-initiated additional motor tasks and additional weight) for each of the four exercises is determined by an experienced therapist at the beginning of the intervention for all participants in the intervention group. The therapist in charge rates the participants’ performance accuracy at level one and derives a starting level. Performance accuracy is thereby standardized rated based on the axis and plane alignment (extremities, trunk) during motion, movement goal (endpoint) accuracy and no loss of balance during motion or single movements order. The starting level is defined as the highest level in which this accuracy can be reached. This level (including the number and type of self-initiated additional motor tasks and additional weight) is further adaptive and may be corrected in both directions (increase or even decrease in level) continuously by the therapist during the centre-based phase. The goal for the intervention is to increment by one level once a week until the maximum level 12 is reached.

The intervention group patients receive individualized exercise counselling for home-based training as follows: at the end of the centre-based trainings, the starting level for the home-based phase is determined (therapist in collaboration with the participant). In addition, the goal level for the home-based phase is determined. Each subjects’ goal level and the periodization to reach this level is subsequently recorded on the training log. Intervention group subjects were additionally encouraged to contact the counselling exercise therapist by phone, e-mail or in person at any time during the home-based intervention. Final therapy individualization may be accomplished by using a standardized procedure to detect potential non-responder and/or non-complier. This detection is based on cutoff values which will be determined from the studies’ questionnaires. If a potential non-responder and/or non-complier is detected, each study site is recommended to implement an additional behavioural module to the intervention. This module and its empirical funding is described in detail elsewhere [34].

Control group participants do not receive any additional intervention in/from one of the study centres but may proceed with their regular care (e.g. physiotherapy or manual therapy) or additional interventions (the same applies to the intervention group) if they are engaged in one at the time of study inclusion. The intervention within medicine in spine exercise (MiSpEx) thus can be seen as additive.

#### Therapy monitoring

During the stationary centre-based phase, a standardized training log is completed by the therapist, documenting the exercise level and the (if applied) additional weight. To monitor home training compliance, intervention group patients are asked to fill in an exercise log during the home-based training (between M2 and M3).

### Statistical analysis

Data is collected and analysed centrally after assessment completion. Following visual and physiological range plausibility control, a gain score analysis (change score analysis) using the mean difference between M1 and M3 for between-group differences of the primary outcome is performed.

Hypotheses testing for all other measurement times and secondary outcomes are performed using particular fitting regressional and variance analytical testing. Potential mediators and confounders like the amount of physical activity, psychosocial scores, age, gender, initial pain level and body weight will be analysed using multiple regression mediating analyses [35] or included into variance analyses as covariates. For mediation analyses, total as well as direct and indirect effects are calculated. Sobel testing and 95 % confidence interval (CI) bias corrected bootstrapping (n_sub-samples_ = 10,000) are conducted for indirect effect quantification [36–38].

All statistical procedures are executed after the examination of underlying assumptions for parametric or rather non-parametric hypotheses testing. Due to the explorative analyses of the secondary outcomes, no alpha-error adjustment is performed. To increase statistical power, missing values are multiple imputed using standardized procedures [39] and only following (in case of psychometric scores) specific test manuals. The one-sided a priori level of significance is set at α = 0.025 for all statistical analyses.

## Discussion

Primary aim of the study is to test the feasibility and efficacy of individualized sensorimotor training interventions for the treatment of nonspecific low back pain. To do so, a standardized and practically relevant diagnostic assessment and a randomized controlled single-blind intervention design is used.

The intervention consists of sensorimotor exercises for core/trunk stability with additive perturbation elements [40] in highly individualized yet guided group therapy sessions. Although there is compelling evidence for the relevance of exercise elements in the therapy of chronic nonspecific low back pain [11, 14], only limited data provides solid information on dose-response and sensorimotor training individualization. Moreover, the biopsychosocial effects of such interventions are yet to be unravelled.

A strength of the intervention implemented is the symbiosis of the hypothesized large and clinically relevant effect and the short exercise duration as well as the low use of resources for both the home- and centre-based intervention [11], making it a cost-effective intervention. The individualized diagnostics and exercises are of further value. The heterogeneity of the study population may become a limitation for data interpretation and practical relevance deduction however. To avoid or minimize this potential bias, mediation analyses, multiple regressions and analyses including potential co-variables are performed. Together with the randomized study design and the resulting respect of unknown confounders, considering these known and suggested confounders provides a sufficient statistical power. A common limitation in exercise trials is the limited possibility to blind the participants. This limitation is increased by the subjective assessment of pain and pain-related function. To reduce the risk of bias as much as possible, all investigators are blinded to intervention allocation.

A suggested underlying mechanism for the hypothesised effect is mostly seen in the analgesic effect of exercise. Exercise releases beta-endorphin both spinal and supraspinal by activating μ-opioid receptors [41]. Following that, an acute sensible decrease in pain is felt. On the long term, exercise and, in particular, sensorimotor or motor control training increases the functional capacity of all involved tissues [42].

Our study provides further evidence for the efficacy or non-efficacy of motor control exercise targeting neuromuscular factors like kinematics, strength, postural control and pain [11]. Beyond that, the results and practical relevance of our study will be of importance not only for researchers and policy makers but also for patients suffering from nonspecific low back pain. On the one hand, the target group diagnostics we implement are of relevance for the planning and longitudinal screening of our individualized motor control exercise. On the other hand and if of clinical relevance, our motor control exercise provides a as far as possible individualized intervention, characterized by low effort and cost.

### Trial status

Participant recruiting started January 2016. At the time of manuscript submission, we had included 142 participants into the study with no participant having completed the intervention. Screening completion is anticipated to be April 2017, measurement completion April 2018 and study completion is expected to be December 2018.

## Appendix A. Study sites, local principal investigators and scientific responsibilities

The MiSpEx-Network multicenter study’s sites are:

Department of Orthopaedics and Trauma Surgery, Heidelberg University Hospital, Heidelberg, Germany; Prof. Dr. Marcus Schiltenwolf.

Department of Sports Medicine and Sports Nutrition, Ruhr-University Bochum; Prof. Dr. Petra Platen.

Institute of Sports Orthopaedics, Schön Klinik München Harlaching; Dr. Christian Schneider.

Department of Sports Medicine, Goethe University Frankfurt, Frankfurt am Main, Germany; Prof. Dr. Dr. Winfried Banzer; Prof. Dr. Lutz Vogt.

University Hospital Carl Gustav Carus at Technical University Dresden, Dresden, Germany; Dr. Heidrun Beck.

University Outpatient Clinic, Center of Sports Medicine, University of Potsdam, Potsdam, Germany; Prof. Dr. Frank Mayer; Principal Investigator.

## Additional file

Additional file 1: Figure S1. Schedule of enrolment, interventions, and assessments. (DOC 41.0 kb)

## Abbreviations

AEQ-PPS: Avoidance‐Endurance Questionnaire
BSSS: Berlin Social Support Scales
CI: confidence interval
CMJ: counter movement
CoP: center of pressure; static balance
COP: centre of pressure
CPG: Chronic Pain Grade Questionnaire
CRT: Chair Rise Test
DRKS: German Clinical Trials Register
F: force/strength
FABQ: Fear-Avoidance Belief Questionnaire
HADS: Hospital Anxiety and Depression Scale
ICC: intraclass correlation coefficient
IPAQ: International Physical Activity Questionnaires
LE: life events
M1: M2, M3, M4, M5, visits 1–5
MiSpEx: Medicine in Spine Exercise
MIVF: maximal isometric voluntary force
N: number
NRS: numeric rating scale
ROM: range of motion/kinematic analyses
RQ-2: relationship questionnaire
SES: socio-economic status
SMT: sensorimotor training
TICS: Trier Inventory for Chronic Stress
TuG: Timed Up and Go Test
