# Erratum to: Medicine in spine exercise (MiSpEx) for nonspecific low back pain patients: study protocol for a multicentre, single-blind randomized controlled trial

**DOI:** 10.1186/s13063-017-1814-x

**Published:** 2017-02-06

**Authors:** Daniel Niederer

**Affiliations:** Department of Sports Medicine, Goethe University Frankfurt, Frankfurt am Mai, Germany

## Erratum

Unfortunately, the original version of this article [1] contained an error. The correct term can be found below:

Sufficient test–retest reliability and validity (r^2^ > 0.97; RMSE = 4.1°; p < 0.001) of this measurement system for lumbar spine kinematics has recently been demonstrated [31].
