# Supplementary material for: Medicine in spine exercise (MiSpEx) for nonspecific low back pain patients: study protocol for a multicentre, single-blind randomized controlled trial
Source: Trials. 2016 Oct 20;17:507. doi: 10.1186/s13063-016-1645-1 (PMC5072341; doi:10.1186/s13063-016-1645-1)
Supplement: Additional file 1: Figure S1. — Schedule of enrolment, interventions, and assessments. (DOC 41.0 kb) [file 13063_2016_1645_MOESM1_ESM.doc]

**Schedule of enrolment, interventions, and assessments.**

|  | **STUDY PERIOD** | | | | | |
| --- | --- | --- | --- | --- | --- | --- |
| **TIMEPOINT**** | ***-t_1_*** | ***t_1_ &***  ***allocation*** | ***t_2_*** | ***t_3_*** | ***t_4_*** | ***t_5_***  ***t_x_*** |
| **ENROLMENT:** |  |  |  |  |  |  |
| **Recruiting** | X |  |  |  |  |  |
| **Eligibility screening** | X |  |  |  |  |  |
| **Informed consent** | X |  |  |  |  |  |
| **Allocation** |  | X |  |  |  |  |
|  |  |  |  |  |  |  |
| **INTERVENTIONS:** |  |  |  |  |  |  |
| ***SMT center-based*** |  |  |  |  |  |  |
| ***SMT home-based*** |  |  |  |  |  |  |
| ***Control*** |  |  |  |  |  |  |
| **ASSESSMENTS:**  See Figure 1 for details |  | M1 | M2 | M3 | M4 | M5 |
